# 99 Retrospective Review of Pediatric Fluid Resuscitation at a Large Burn Center

**DOI:** 10.1093/jbcr/irad045.072

**Published:** 2023-05-15

**Authors:** Stacey Richerbach, Natalie Kesler, Suzanne Osborn, Van Dobbe, Claudia Islas, Karen Richey, Kevin Foster, Devin Nikjou

**Affiliations:** Arizona Burn Center at Valleywise Health, Phoenix, Arizona; AZ Burn Center at Valleywise Health, Phoenix, Arizona; AZ Burn Center at Valleywise Health, Phoenix, Arizona; AZ Burn Center at Valleywise Health, Phoenix, Arizona; AZ Burn Center at Valleywise Health, Phoenix, Arizona; Arizona Burn Center Valleywise Health, Phoenix, Arizona; AZ Burn Center at Valleywise Health, Cave Creek, Arizona; AZ Burn Center at Valleywise Health, Phoenix, Arizona; Arizona Burn Center at Valleywise Health, Phoenix, Arizona; AZ Burn Center at Valleywise Health, Phoenix, Arizona; AZ Burn Center at Valleywise Health, Phoenix, Arizona; AZ Burn Center at Valleywise Health, Phoenix, Arizona; AZ Burn Center at Valleywise Health, Phoenix, Arizona; Arizona Burn Center Valleywise Health, Phoenix, Arizona; AZ Burn Center at Valleywise Health, Cave Creek, Arizona; AZ Burn Center at Valleywise Health, Phoenix, Arizona; Arizona Burn Center at Valleywise Health, Phoenix, Arizona; AZ Burn Center at Valleywise Health, Phoenix, Arizona; AZ Burn Center at Valleywise Health, Phoenix, Arizona; AZ Burn Center at Valleywise Health, Phoenix, Arizona; AZ Burn Center at Valleywise Health, Phoenix, Arizona; Arizona Burn Center Valleywise Health, Phoenix, Arizona; AZ Burn Center at Valleywise Health, Cave Creek, Arizona; AZ Burn Center at Valleywise Health, Phoenix, Arizona; Arizona Burn Center at Valleywise Health, Phoenix, Arizona; AZ Burn Center at Valleywise Health, Phoenix, Arizona; AZ Burn Center at Valleywise Health, Phoenix, Arizona; AZ Burn Center at Valleywise Health, Phoenix, Arizona; AZ Burn Center at Valleywise Health, Phoenix, Arizona; Arizona Burn Center Valleywise Health, Phoenix, Arizona; AZ Burn Center at Valleywise Health, Cave Creek, Arizona; AZ Burn Center at Valleywise Health, Phoenix, Arizona; Arizona Burn Center at Valleywise Health, Phoenix, Arizona; AZ Burn Center at Valleywise Health, Phoenix, Arizona; AZ Burn Center at Valleywise Health, Phoenix, Arizona; AZ Burn Center at Valleywise Health, Phoenix, Arizona; AZ Burn Center at Valleywise Health, Phoenix, Arizona; Arizona Burn Center Valleywise Health, Phoenix, Arizona; AZ Burn Center at Valleywise Health, Cave Creek, Arizona; AZ Burn Center at Valleywise Health, Phoenix, Arizona; Arizona Burn Center at Valleywise Health, Phoenix, Arizona; AZ Burn Center at Valleywise Health, Phoenix, Arizona; AZ Burn Center at Valleywise Health, Phoenix, Arizona; AZ Burn Center at Valleywise Health, Phoenix, Arizona; AZ Burn Center at Valleywise Health, Phoenix, Arizona; Arizona Burn Center Valleywise Health, Phoenix, Arizona; AZ Burn Center at Valleywise Health, Cave Creek, Arizona; AZ Burn Center at Valleywise Health, Phoenix, Arizona; Arizona Burn Center at Valleywise Health, Phoenix, Arizona; AZ Burn Center at Valleywise Health, Phoenix, Arizona; AZ Burn Center at Valleywise Health, Phoenix, Arizona; AZ Burn Center at Valleywise Health, Phoenix, Arizona; AZ Burn Center at Valleywise Health, Phoenix, Arizona; Arizona Burn Center Valleywise Health, Phoenix, Arizona; AZ Burn Center at Valleywise Health, Cave Creek, Arizona; AZ Burn Center at Valleywise Health, Phoenix, Arizona; Arizona Burn Center at Valleywise Health, Phoenix, Arizona; AZ Burn Center at Valleywise Health, Phoenix, Arizona; AZ Burn Center at Valleywise Health, Phoenix, Arizona; AZ Burn Center at Valleywise Health, Phoenix, Arizona; AZ Burn Center at Valleywise Health, Phoenix, Arizona; Arizona Burn Center Valleywise Health, Phoenix, Arizona; AZ Burn Center at Valleywise Health, Cave Creek, Arizona; AZ Burn Center at Valleywise Health, Phoenix, Arizona

## Abstract

**Introduction:**

Foundational approaches to burn fluid resuscitation were largely developed with the adult patient in mind. Experience has shown, however, that physiologic nuances between adults and children necessitate an adjusted approach to resuscitation for pediatric patients. As practice has advanced to account for these differences, our burn center sought to evaluate efficacy of resuscitation for our pediatric patient population. The purpose of this study was to examine pediatric fluid resuscitation including trends and complications, and to establish a baseline for future quality improvement initiatives.

**Methods:**

This was a retrospective review of pediatric patients <14 years of age treated with resuscitation in a burn center between 2017 and 2021. Descriptive statistics were calculated.

**Results:**

Over a five-year period, 42 pediatric patients were admitted with a burn total body surface area (TBSA) >20%, of which 23 met inclusion/exclusion criteria. Average admission weight was 31.8 kg and average TBSA of 39%. Contributing mechanisms of injury were flame (61%), scald (35%) and chemical (4%). Concomitant inhalation injury was diagnosed in 17% of patients. Average hourly fluid resuscitation volume was 6.9 mL/kg/TBSA with a mean resuscitation duration of 25.7 hours. Total volume administered throughout resuscitation exceeded recommendations per the Parkland formula for 83% of the sample. Continuous albumin was administered for 74% of patients and was initiated on average at 10 hours post-injury. Boluses of lactated ringer’s and albumin were administered for 30% and 9% of patients, respectively. Urine output for the pediatric patients averaged 60 mL/hr with a mean low optimal urine output of 15.9mL/hr and high optimal output of 25.5 mL/hr. Adverse events occurring during fluid resuscitation included temperature < 36^◦^C (35%), vasopressor administration (9%), intra-abdominal pressure >12 (13%) and/or >19 (9%), and peripheral vascular pulses < +1 (61%). Prophylactic escharotomy was performed for 35%. Of note, none of the patients were diagnosed with compartment syndrome nor abdominal compartment syndrome and none required abdominal laparotomy. One patient (4%) was diagnosed with acute respiratory distress syndrome. Continuous renal replacement therapy (CRRT) was not utilized for any pediatric patients during or post-resuscitation, compared to 34% of adults resuscitated at the same center. No patients expired in the initial 48 hours post-burn. The overall survival rate for this population was 91.3%, with 87% discharged direct to home.

**Conclusions:**

Resuscitation volumes for pediatric patients in a large burn center have exceeded historically accepted rates for resuscitation. Despite the divergence from traditional practice, associated complications remain low, and outcomes have been favorable.

**Applicability of Research to Practice:**

Further research is needed to establish benchmarks for pediatric fluid resuscitation.

